# Malaria-related mortality based on verbal autopsy in an area of low endemicity in a predominantly rural population in Ethiopia

**DOI:** 10.1186/1475-2875-6-128

**Published:** 2007-09-21

**Authors:** Wakgari Deressa, Mesganaw Fantahun, Ahmed Ali

**Affiliations:** 1 School of Public Health, Addis Ababa University PO Box 9086, Addis Ababa, Ethiopia

## Abstract

**Background:**

Although malaria is one of the most important causes of death in Ethiopia, measuring the magnitude of malaria-attributed deaths at community level poses a considerable difficulty. Nevertheless, despite its low sensitivity and specificity, verbal autopsy (VA) has been the most important technique to determine malaria-specific cause of death for community-based studies. The present study was undertaken to assess the magnitude of malaria mortality in a predominantly rural population of Ethiopia using VA technique at Butajira Rural Health Programme (BRHP) Demographic Surveillance Site (DSS).

**Methods:**

A verbal autopsy was carried out for a year from August 2003 to July 2004 for all deaths identified at BRPH-DSS. Two trained physicians independently reviewed each VA questionnaire and indicated the most likely causes of death. Finally, all malaria related deaths were identified and used for analysis.

**Results:**

A verbal autopsy study was successfully conducted in 325 deaths, of which 42 (13%) were attributed to malaria. The majority of malaria deaths (47.6%) were from the rural lowlands compared to those that occurred in the rural highlands (31%) and urban (21.4%) areas. The proportional mortality attributable to malaria was not statistically significant among the specific age groups and ecological zones. Mortality from malaria was reckoned to be seasonal; 57% occurred during a three-month period at the end of the rainy season between September and November. About 71% of the deceased received some form of treatment before death, while 12 (28.6%) of those who died neither sought care from a traditional healer nor were taken to a conventional health facility before death. Of those who sought treatment, 53.3% were first taken to a private clinic, 40% sought care from public health facilities, and the remaining two (6.7%) received traditional medicine. Only 11.9% of the total malaria-related deaths received some sort of treatment within 24h after the onset of illness.

**Conclusion:**

The results of this study suggest that malaria plays a considerable role as a cause of death in the study area. Further data on malaria mortality with a relatively large sample size for at least two years will be needed to substantially describe the burden of malaria mortality in the study area.

## Background

Malaria is one of the most important causes of morbidity and mortality in many tropical countries. About 90% of the deaths are believed to occur in sub-Saharan Africa (SSA) [1]. Mortality due to malaria is more prevalent among children under-five years of age in highly endemic areas. As a result, most of the already few studies on malaria mortality have focused on childhood malaria in areas of intense transmission [2-6]. Consequently, little is known about the effect of malaria on population exposed to unstable malaria, giving rise to epidemics [7]. In areas of seasonal and unstable malaria transmission like in Ethiopia [8], all age groups of the population are at risk of death, particularly during epidemics, due to a lack of acquired immunity [9,10].

As in many developing countries in SSA, the health information system in Ethiopia is generally weak and provides unreliable estimate on causes of death [11]. This weakness in the system also inevitably affects data on malaria-related mortality. The validity of hospital-based data is also severely limited and represents only a fraction of the actual burden since a considerable proportion of malaria deaths occur at home [3]. Despite its low sensitivity and specificity [12], verbal autopsy (VA) is currently the only alternative approach that has been used to determine malaria-specific cause of death for community-based studies in many of the endemic countries [2-5]. VA is a method of ascertaining causes of death from the circumstances, events, symptoms and signs of illness experienced by the deceased before death reported by bereaved families or close relatives. The cause of death is established by reviewing the VA questionnaire by one or more physicians, or from a set of algorithms. Other methods of interpreting VA results for malaria, such as adjusting the proportion of deaths attributable to malaria for the varying sensitivity and specificity, have also been inconclusively attempted [13,14].

As a means of promoting an effective and sustainable malaria control and to influence policy decisions, monitoring malaria mortality is one of the critical areas for evaluating the progress and impact of interventions. Such information is partly obtained from community-based longitudinal demographic surveillance of a population through correct identification of causes of death [15]. Fortunately, such surveillance systems are now operational in many African countries including Ethiopia in sentinel sites, and these sites have joined together in the INDEPTH (International Network of field sites with continuous Demographic Evaluation of Populations and Their Health in developing countries) network (INDEPTH Network) [16].

The aim of this study was to assess the magnitude of malaria mortality in a predominantly rural population of Ethiopia using VA technique at Butajira Rural Health Program (BRHP) Demographic Surveillance Site (DSS). The findings from this studywould help to understand the magnitude of malaria mortality, and provide evidence for monitoring the impact of malaria prevention and control interventions towards the National Roll Back Malaria goals [Five-year strategic plan unpublished documents, 2001, 2006].

## Methods

### Study area and population

This study was carried out in Meskan and Mareko district (commonly referred as Butajira) in the south-central part of Ethiopia. The district is administratively located in the Gurage Zone, with its capital, Butajira Town, at a distance of 130 km south of Addis Ababa and 50 km west of Zeway, the base of the Rural Community Health Training Programme for under graduate medical students of the Faculty of Medicine at Addis Ababa University (AAU). In 2003, the population of the district was estimated at 300,000 and about 87% were living in rural areas. With altitudes ranging from 1,500 m to 3,500 m above sea level, the district has diverse climatic conditions, ranging from lowlands to highland mountainous areas, and with multi-ethnic groups inhabiting it [17].

Most inhabitants of the rural population are mainly subsistence farmers. Enset (*Enset ventricosum*) and maize are the major staple foods in most parts of the district. To a lesser extent, the people also complement their livelihood by raising livestock. Pepper and khat (*Catha edulis*) are the main cash crops in the area. The district has one Hospital, one health centre, three health stations, two Malaria Control Centres, 12 health posts and 18 registered non-governmental health care providers. Most of these health facilities are concentrated in Butajira town. About 77% of the population is illiterate, although this figure is higher for the rural population.

Infectious diseases, maternal and child health problems have been the most important health problems of the district. Malaria is locally known in the district by the name *weba*. According to three years (2003–2006) consecutive annual reports of Butajira Hospital, malaria was the leading cause of death contributing 53.8%, 32.6% and 18.5% of the deaths, respectively (Butajira Hospital unpublished annual reports).

### Butajira Rural Health Programme (BRHP)

The BRHP-DSS was established in 1986 as a collaborative research project between the Department of Community Health at the Faculty of Medicine, AAU in Ethiopia and the Department of Public Health and Clinical Medicine, Umeå University, Sweden [17]. The BRHP-DSS has a community-based continuous registration of vital and migratory events such as birth, death, marriage, migration and internal mobility at a household level in 10 randomly selected villages (*kebeles*) with a population of about 40,000 [17]. Nine of the ten *kebeles *are rural. The status of each member of the study households is checked on a quarterly basis by village-based high school graduate enumerators.

### Verbal autopsy procedure

The present study was part of a large study that examined the patterns of all-causes of mortality by age and sex at the BRHP-DSS [18]. The WHO and INDEPTH Network  [19] standardized VA questionnaire was adapted and translated into *Amharic*, the official language, for data collection. In short, the questionnaire assessed the identity of the deceased and established the sequence of events leading to death including symptoms and signs of the illness before death. Verbal autopsy was carried out for a year from August 2003 to July 2004. All deaths identified through the quarterly visits were notified by the DSS regular supervisors to the interviewers. Five interviewers specifically trained for the study collected the data and two *kebeles *were assigned for each of them. For every case of death during the study period, the bereaved family or heads of the households were interviewed 45–60 days after death to avoid the intense period of mourning in the study area.

Two trained physicians with local experience independently reviewed each VA questionnaire and indicated the most likely causes of death. None of the physicians were aware of the study on malaria-specific cause of death. They were instructed to identify multiple underlying causes if appropriate. Most of the VA forms were coded only with one main cause of death. In some, the physicians reported two causes of death. When there were discordances between the physicians about the cause of death, they met and discussed to reach on a consensus. The physicians were also provided with an abridged list of ICD-9 (International Classification of Diseases, 9^th ^version) to classify causes of death [20].

### Data analysis

The data on the VA questionnaire and the causes of death assigned by the physicians were coded and entered into SPSS version 11 (SPSS, Chicago, IL, USA) statistical software package for analysis. Frequencies, percentages and cross-tabulations were used to compare malaria deaths between sexes, age groups, ecological zones, pattern of deaths over a period of study and treatment seeking behaviour before death.

### Ethical clearance

The study had ethical approval from the Ethical Committee of the Faculty of Medicine at AAU and the Ethiopian National Ethics Committee as a component of the BRHP research. Verbal informed consent was obtained from all the bereaved families or heads of households of the deceased after explaining the purpose and the procedures of the study.

## Results

With a mid-year population of 49,825 (50.3% females) during the one-year study period between August 2003 and July 2004, a total of 370 deaths and 1634 births were registered in the area. There were 190 under-five deaths (51.4%); among those, 134 (70.5%) occurred in those less than one year, giving the under-five and infant mortality rates of 116.3 and 82 per 1000 live births, respectively. There were 56 deaths in children 1 to 4 years old. The crude mortality rate for the year was estimated at 7.4 per 1,000.

Of the total recorded deaths, VA was successfully carried out in 325 deaths (88%), but due to field staff turn over and late notification of deaths the review was not available for 12% of the cases. In all, 42 (13%) of the 325 deaths reviewed from VA were attributed to malaria. In 26 cases (61.9%), malaria was the only cause of death assigned. In addition to malaria, other causes of death were also assigned for each of the remaining 16 deaths (39.1%).

Overall, deaths among under-five children accounted for 14 (33.3%) of all malaria related deaths; of whom, three (21.4%) were among infants and 11 (78.6%) among children 1 to 4 years old. Children between 5–9 years accounted only for 1 (2.4%), 10–14 years old accounted for three cases (7.1%). The proportional mortality attributable to malaria is 9% in 0–4 year-old children, 28.6% in 5–14 year-old children and 15% in ≥ 15 year-old adults (Table 1). These age specific variations in proportional mortality attributable to malaria between age groups are not statistically significant (P-value = 0.054).

**Table 1:** Age and sex composition of malaria deaths compared to all causes as indicated by verbal autopsy, BRHP DSS, 2003/04.

**Age category (years)**	**Deaths from all causes (%)**	**Malaria deaths by sex**		
		
		**Male, No.**	**Female, No.**	**Total (%)**
0–4	154 (47.4)	5	9	14 (9)
5–9	8 (2.5)	0	1	1 (12.5)
10–14	6 (1.8)	2	1	3 (50.0)
15–19	13 (4.0)	2	2	4 (30.8)
> 19	144 (44.3)	10	10	20 (13.9)

Total, No. (%)	325 (100)	19 (45.2)	23 (54.8)	42 (13.0)

Of the total malaria related deaths, 23 (54.8%) occurred among females. The majority of malaria-related deaths (47.6%) were from the rural lowlands compared to those occurred in the rural highlands (31%) and urban (21.4%) areas (Table 2). The proportional mortality attributable to malaria between the ecological zones was 14.3%, 10% and 16.7% in rural lowland, rural highland and urban highland, respectively, showing statistical insignificance (P-value = 0.377). Mortality from malaria was seasonal; 24 of 42 deaths (57%) occurred during a three-month period at the end of the rainy season during September to November, followed by a minor peak during May-June.

**Table 2:** Malaria-related mortality from verbal autopsy in relation to population and all other causes of deaths by ecological zone, BRHP DSS, 2003/04.

		**Cause**		
			
**Ecological zone**	**Population**	**Malaria, No. (%)**	**Other, No. (%)**	**Total, No. (%)**
Rural lowland	16 680	20 (14.3)	120 (85.7)	140 (43.1)
Rural highland	19 346	13 (10.0)	118 (90.0)	131 (40.3)
Urban highland	13 799	9 (16.7)	45 (83.3)	54 (16.6)

Total, No. (%)	49 825	42 (13.0)	283 (87.0)	325 (100)

It was reported that 40 of 42 deaths (95.2%) were sick within the last three months before death. The family first recognized the onset of illness within the first 24 h of the onset of illness for 26 (50%) of the patients who succumbed up for death, 1–3 days for 10 deaths and after three days for 6 deaths. The severity of the illness was asked on a ranking scale and found to be very serious for 31 deaths (73.8%), serious for 3 deaths and not serious for 8 deaths (19.1%).

Thirty (71.4%) of the deceased received some form of treatment before death. However, 12 (28.6%) of those who died neither sought care from a traditional healer nor were taken to a conventional health facility before death. The proportion of patients who received no care before death was higher for rural (38.46%) and urban (33.33%) highlands than the rural lowland areas (20%). Pattern of health service utilization before death is shown in Table 3. Of those who sought treatment, 16 (53.3%) were first taken to a private clinic, and the remaining 12 (40%) were taken to public health facilities such as hospital, health center, and health station/post. The most common sources of second-line treatment were hospital (40%), private clinic (30%) and health center (20%).

**Table 3:** Patterns of visit to various sources of treatment among malaria deaths identified by verbal autopsy, BRHP DSS, 2003/04.

Treatment sources	First choice, n (%)	Second choice, N (%)	Third choice, n (%)
Private clinic	16 (53.33)	6 (30.00)	2 (50.00)
Hospital	4 (13.33)	8 (40.00)	2 (50.00)
Health center	3 (10.00)	4 (20.00)	0
Health station/post	5 (16.67)	1 (10.00)	0
Pharmacy/drug shop	0	1 (10.00)	0
Traditional medicine	2 (6.67)	0	0

	30 (100.00)	20 (100.00)	4 (100.00)

Among 30 of 42 who sought some sort of treatment before death, 31.3% visited or got treatment from one source, 53.3% visited two sources of treatment and 13.3% sought it from three sources (Table 3). Most of those (66.7%) who got help from the first source of treatment continued to seek care from the second source of care as their condition remained unimproved by the first action; of whom 4 (20%) further switched to the third source of treatment. Among the 12 patients who had never sought care, 3 died while on the way to seek care from health facility. For three of the deceased financial problem was reported as a barrier for not seeking treatment, the illness was thought to be mild for two, the illness was very brief (acute) for the other two, and the reason could not be known for one death.

The main symptoms reported by the bereaved families or close relatives of 31 (73.8%) of the 42 deaths were high fever (61.3%), headache (41.9%), vomiting (22.6%), cough (12.9%), chills/shivering (9.7%), diarrhea (9.7%), chest pain (9.7%), epigastric burning (9.7%), loss of appetite (6.5%), convulsion (6.5%), discoloration of urine (6.5%), grunting (6.5%) and thirsty (3.2%).

Among those who visited health facilities, five were referred to a higher level of health care. However, only two of the referred patients visited the said higher level of health care facility. After the visit of the first health care provider, the condition of the 19 (63.3%) patients was not improved or even worsened after receiving the initial treatment. Respondents were also asked to state the duration between the recognition of the illness and treatment seeking for the deceased. Only 11.9% of the total malaria-related deaths received some sort of treatment within 24 h after the onset of illness (Table 4).

**Table 4:** Promptness of treatment seeking after recognition of malaria illness before death, BRHP DSS, 2003/04.

**Days of treatment seeking after illness onset**	**Frequency, n**	**Percentage**
Treatment not sought	12	28.57
Within 24 hours	5	11.90
1–2 days	12	28.57
3–4 days	8	19.06
≥ 5 days	5	11.90

Total	42	100

## Discussion

This community-based study attempted to determine the importance of malaria as a cause of death using a VA technique in a predominantly rural community residing in an area of seasonal malaria transmission in Ethiopia [8,21]. It also assessed treatment-seeking behaviour preceding the death. In the study area, malaria was responsible for 13% of the total deaths reviewed for VA during the one-year period of study, giving the overall malaria mortality rate of 0.84 per 1,000 per year. In the previous VA study in the same area, 0.6 deaths per 1,000 person-years was attributed to malaria [22].

The major causes of death at BRHP-DSS are acute cardiac, acute infection, diarrhea, tuberculosis, malignancy, HIV/AIDS and maternal causes [18]. Deaths due to communicable and non-communicable diseases accounted for about 60% and 25% of the total adult (15–49 years) deaths reported from 1995 to 1999 in the same area [23]. In the later study, acute febrile illnesses, mainly presumed to be malaria, were responsible for about 25% of all deaths.

Unlike areas with perennial malaria transmission where more malaria deaths occur among children under-five years of age [4,5], the present findings indicate that malaria was responsible for deaths among all age groups of the population. The age specific proportional mortality attributable to malaria showed no statistically significant difference. This can be due to small samples or due to misclassification error of VA technique. Previous studies conducted in the area indicated that malaria accounted for 14% of all deaths in under-five children [24]. In The Gambia, where malaria is seasonal, it was identified as a probable cause of 14% of deaths in under-five children [3]. In areas where malaria is holoendemic, malaria was identified as a cause of more than half of the deaths in children less than five years of age [4,5].

Malaria transmission in the study area is seasonal and the population lacks acquired immunity, as it is true for most parts of the country [8,21]. Over the one-year study there were more malaria deaths during September-November, following the heavy rainfall in summer. The second minor peak in the number of deaths occurred during May-June, after the short rains in March and April. It is to be expected that the number of malaria deaths in the dry season would be smaller than during or after the rainy season. The findings substantiate the importance of malaria as a major health problem in BRHP-DSS, and indicate the similarity with the national pattern of malaria transmission where high number of malaria cases or deaths occur during the major transmission season from September through December and the minor from May to June [8,21].

Malaria proportionate mortality was similar in the three ecological zones considered in this study. Although the total number of deaths was small to make valid statistical inferences, the similarity in the proportional mortality attributable to malaria between the ecological zones is probably due to the effect of misclassification of causes of death of the VA method rather than the real lack of variability between the zones.

In the same study area, studies revealed higher overall mortality rates in the lowlands compared to highland areas [25]. These variations may partly be explained by the high prevalence of malaria in the lowland areas. The lowland areas under BRHP-DSS are located at an altitude between 1,500 m and 1,750 m above sea level (asl), while the highlands range between 1,750 m and 2,300 m asl [17]. Areas below 2,000 m asl in Ethiopia are generally classified as malarious and those between 2,000 and 2,500 m asl are periodically affected with malaria epidemics related to weather anomalies [26].

About 29% of those who died were taken neither to a health facility nor to a traditional healer before death. In addition, the health status before death of most of those who died deteriorated after an initial visit to the first-line of care, presumably due to poor quality of care received from the care provider or delay in seeking treatment. In a community-based cross-sectional mortality survey in Tigray Region, about 45% of under-five children who probably died due to malaria did not seek care either from a health facility or a CHW before death [27]. In The Gambia, most malaria deaths among children occurred within 2–3 days after onset of illness and most of the children who died had not sought treatment [3]. These findings suggest that mortality from malaria could be reduced in Ethiopia by making effective antimalarial drugs available closer to home through the currently advocated and scaled-up health extension programme.

Very few studies have been conducted to study causes of death across all ages using VA [18,22,28,29]. Most studies on malaria as a cause of death have focused on childhood malaria [2,4,5] and little is known about malaria mortality among older children and adults in malaria endemic areas in Africa [7]. Consequently, comparative data on health care utilization among adults before death are very limited for review, but could be speculated that it wouldn't be better than treatment seeking for under-five children. The main reasons for under-utilization of health services include poor geographical or financial access, workload of the family, the expectation that the illness would terminate itself and poor knowledge about the need for the early treatment [30].

Although public health facilities are assumed to have better organization and institutional capacity, the present findings indicate that more than half of those who died due to malaria made first visit to a private clinic for treatment. In other words, people in rural areas have little access to public health facilities in addition to the inefficient services rendered by the public health-care system [31]. The reasons for the high utilization of private clinics included the perception that they provide high quality services and give much attention compared to the public services, despite their high cost of treatment.

Assessing the importance of malaria as a cause of death in a predominantly rural community is a challenge. Although verbal autopsy for malaria has been shown to have a low level of sensitivity and specificity [12], it is the only method currently available to assess malaria mortality at community level in most malaria endemic areas in Africa where most deaths occur at home without medical attention. The WHO recommends that verbal autopsies can be used to assess malaria-specific cause of death in a community-based longitudinal DSS [15,22].

In spite of the limitations of the VA technique, the questionnaire used in this study was prepared and standardized by WHO and INDEPTH Network to assess all cause-specific mortality in all ages, and adapted to the present setting. Since the interviewers applied the questionnaires for all deaths, any over- or under-estimation of malaria deaths is not a concern. However, it should be noted that the study was conducted during a single year and does not reveal the seasonality of malaria from year-to-year, requiring the need for caution in drawing general conclusions from the findings. In addition, there is a potential risk of misclassification about the causes of death where the sensitivity and specificity of the VA technique is relatively low for assessing malaria mortality [12].

The study site in BRHP-DSS encompasses areas of low and high altitude further characterized by diverse cultural and ethnic variations. Therefore, extrapolation of the results to other rural parts of Ethiopia with different levels of malaria endemicity should be cautiously considered. More findings are needed to find out whether similar results from BRHP-DSS are generalizable to other comparable parts of the country. However, the site allows the observation of a wide range of health and health related problems in the lowland and highland areas as well as from the point of view of diverse ethnic and cultural variations [17].

## Conclusion

The results of this study suggest that malaria plays a considerable role as a cause of death in the BRHP-DSS. Although the findings may serve as the base-line data on the magnitude of the burden of malaria at community level and thus assist in planning of malaria prevention and control interventions, further data on malaria mortality with a relatively large sample size for at least two years will be needed to describe the seasonality of malaria in the study area. In addition, validation of the VA technique according to the prevailing causes of death, particularly malaria, in this setting using Butajira Hospital data as a gold standard would be helpful to justify the use of this technique in Ethiopia.

## Authors' contributions

1. WD participated in the planning and implementation of the study, analyzed the data, drafted and finally revised the manuscript.

2. MF conceived the study, participated in the planning and implementation of the study, participated in data analysis and critically commented on the draft manuscript.

3. AA participated in the planning and implementation of the study, and critically commented on the draft manuscript.

All authors read and approved the final manuscript.
